# Management of first metatarsophalangeal joint osteoarthritis by physical therapists and podiatrists in Australia and the United Kingdom: a cross-sectional survey of current clinical practice

**DOI:** 10.1186/s13047-020-0382-6

**Published:** 2020-03-13

**Authors:** Kade L. Paterson, Rana S. Hinman, Hylton B. Menz, Kim L. Bennell

**Affiliations:** 1 Centre for Health, Exercise and Sports Medicine, Department of Physiotherapy, School of Health Sciences, The University of Melbourne, Parkville, Melbourne, Victoria 3010 Australia; 2 School of Allied Health, Human Services and Sport, La Trobe University, Melbourne, Victoria Australia

**Keywords:** Foot osteoarthritis, Allied health care, Podiatry, Physiotherapy, Exercise, Orthoses

## Abstract

**Background:**

First metatarsophalangeal (MTP) joint osteoarthritis (OA) is a common and painful problem that causes significant disability. There is limited research on assessment and treatment options, and the efficacy of current management strategies is unknown. The aim of this study was to determine how podiatrists and physical therapists in Australia and the United Kingdom (UK) manage people with first MTP joint OA.

**Methods:**

A survey of podiatrists and physiotherapists was conducted. Potential respondents were recruited through professional representative organisations in Australia and the UK. Participants completed a bespoke online survey regarding the assessment and treatment approaches they most commonly use for patients with first MTP joint OA. Descriptive statistics were calculated and differences between professions compared using chi-square.

**Results:**

Two hundred respondents (*n* = 113 (57%) podiatrists and *n* = 140 (70%) from Australia) completed the survey. Assessment tests were similar between professions and included x-ray (*n* = 151/164; 92%), range of motion (*n* = 127/141; 90%), and a pain scale (*n* = 78/99; 79%). Podiatrists were more likely than physical therapists to discuss over-the-counter medication (42% vs 17%; *p* < 0.001), prescribe orthoses (97% vs 66%; *p* < 0.001), particularly custom orthoses (78% vs 42%; *p* < 0.001), and provide advice on footwear (92% vs 78%; *p* < 0.01) when treating first MTP joint OA. In contrast, physical therapists used more exercise-based approaches to treatment, including exercise therapy (91% vs 34%; *p* < 0.001), increasing general activity (70% vs 49%; *p* < 0.01), and advice to pace activities (83% vs 48%; *p* < 0.001).

**Conclusion:**

Podiatrists and physical therapists use an array of assessment and treatment approaches for people with first MTP joint OA, albeit there is limited evidence to support their clinical utility. Treatment strategies differ between professions, particularly with respect to medication, orthoses and exercise. It is unclear whether these commonly-used strategies improve symptoms associated with first MTP joint OA.

## Background

Osteoarthritis (OA) is a common and painful problem that causes significant disability. The knee and hip are considered the most commonly affected lower limb joints [1], however recent population data showed that symptomatic radiographic OA of the first metatarsophalangeal (MTP) joint affected 7.8% of people aged over 50 years, making it as prevalent as hip OA [2]. First MTP joint OA is debilitating, with nearly three quarters of afflicted individuals describing the pain as “disabling” [2]. People with the condition also experience substantial difficulties performing functional weight-bearing activities, and have significantly worse quality of life compared to those without the condition [3].

Despite its prevalence and disease burden, there is scant evidence to guide treatment of first MTP joint OA. There have only been three randomised clinical trials (RCTs) investigating conservative treatment for the condition [4–6] and only one trial evaluating surgery [7]. In contrast, a recent systematic review and meta-analysis found 97 RCTs for treatment of hip OA [8]. This lack of research on first MTP joint OA makes it difficult for clinicians to base practice on robust evidence, and therefore, to separate effective from ineffective treatments.

Before designing clinical trials to evaluate treatments for first MTP joint OA, a logical first step is to identify assessment and treatment management strategies used by the health professionals to which patients most commonly consult. Although data is lacking for first MTP joint OA, research in people with midfoot OA show they commonly consult with podiatrists (48%), general practitioners (46%), and physical therapists (19%) [9]. To date, no study has investigated how any of these health professionals manage people with OA of the first MTP joint, however, we recently reported that Australian general practitioners manage people with the broader definition of “foot and/or ankle OA” largely using medications [10]. It would also be of interest to assess how podiatrists and physical therapists manage people with first MTP joint OA, as this information on treatment trends would help establish priorities for further research. Therefore, the primary aim of this study was to determine how podiatrists and physical therapists in Australia and the United Kingdom (UK) currently assess and treat people with OA of the first MTP joint. A secondary aim was to compare the assessment and treatment approaches used by the two professions.

## Methods

### Design

This study was a survey of podiatrists and physical therapists in Australia and the UK. The two countries were chosen due to their similar health care systems and the comparable scopes of practice of the two professions in each. Protocols were approved by the institutional Ethics Committee and participants provided informed consent.

### Participants

Between July and September 2015, we contacted the Australian Podiatry Association, Australian Physiotherapy Association, The College of Podiatry (UK), The Chartered Society of Physiotherapy (UK) and our podiatry and physiotherapy clinical and university partners (e.g. hospital department managers, local clinics). Our email explained the purpose of our study and requested the distribution of a link to the study plain language statement and the survey. We also advertised directly to podiatrists and physical therapists in Australia and the UK on Facebook. All podiatrists and physical therapists currently registered and practising in Australia and the UK, and who self-reported managing at least one patient with first MTP joint OA in the previous 6 months, were eligible to participate.

### Procedures

Prior to drafting the survey, we conducted a literature search to identify published assessment and treatment approaches for older people with foot pain and/or symptomatic first MTP joint OA. Given the very limited research on the condition, any approach that was published in English was included. Survey questions and a case vignette of a typical patient with first MTP joint OA were then drafted. Case vignettes, which consist of ‘text, images or other forms of stimuli to which research participants are asked to respond’ [11], are accurate and valid tools for assessing clinical practices [12]. The survey was then completed by the researchers and a small number of clinicians currently treating people with first MTP joint OA to test the survey length, and to check for clarity and errors. Interested participants accessed the online survey either directly, by selecting the link distributed in the study information, or by contacting the lead researcher (KP). The survey was administered online using Survey Gizmo (www.surveygizmo.com; Boulder, CO) and took approximately 15 min to complete.

The survey comprised six main sections. Sections one and two contained a plain language statement outlining the study, and eligibility questions. Section three contained general demographic questions regarding occupation, qualification(s) and year(s) gained, sex, age, employment setting and grade, frequency treating patients with symptomatic first MTP joint OA, and postgraduate training. The remaining sections of the survey all began with the same first MTP joint OA case vignette (Table 1), followed by questions regarding assessment and treatment practices that clinicians may use at some stage over the course of the patient’s management. Of these, section four presented questions regarding specific investigations, physical performance/impairment measures, and tools and questionnaires relevant for the first MTP joint OA case. This section also contained general questions regarding common treatment approaches and practices for the case patient, such as intervention approaches, referrals, and patient advice including analgesia use and weight loss. Section five included questions on specific exercise prescription practices for the first MTP joint OA case patient, and the final survey section contained a series of questions regarding the prescription and types of foot orthoses. For each of these sections, respondents were required to select from a list any items that they felt were important to use when assessing or treating a patient with OA of the first MTP joint. In addition to selecting a response, respondents were also able to make suggestions for additional assessment or treatment items they felt were important but not included in the survey. Responses for each item selected or suggested were then summed to determine the number of respondents who used each approach.

**Table 1:** Case vignette of a typical patient with symptomatic first MTP joint OA

A 63 year-old woman was referred from her general practitioner due to left foot pain, which began insidiously 3 years ago and has steadily worsened over time. Her general practitioner diagnosed her as having first metatarsophalangeal (MTP) joint OA. She is very anxious about the possibility of having foot surgery, feels that her pain is just going to get worse and believes there is nothing she can do to prevent this. She has not had any previous treatment for her foot pain and her health is generally good, although she is overweight, and is on daily medication for hypertension. She is a retired receptionist, lives with her husband and babysits her 4 year-old grandson 2 days per week while her daughter works. Today she rates the intensity of her foot pain as 6 out of 10. Pain is aggravated by twisting and turning, walking on uneven surfaces and wearing shoes with heels or narrow footwear. She is limited in her ability to perform her daily home duties, and can only vacuum for around 10 min before she has to stop. She finds some relief from applying heat, and takes over-the-counter Paracetamol when she needs it, which is around twice per week. Her big toe joint feels stiff first thing in the morning, which eases after approximately 20 min. On examination she has a moderately large and somewhat painful bony exostosis on the dorsal aspect of her left first MTP joint. She does not have a medial exostosis (i.e. bunion). Her first MTP joint range of motion is 35° and it has a hard end feel with some crepitus. She has a moderately pronated foot posture and during gait she exhibits excessive midfoot pronation after heel contact, with minimal resupination during propulsion. She also has reduced left first MTP joint motion during the stance phase of gait accompanied by an early heel rise and excessive knee flexion. No other examination findings are remarkable.	

### Statistical analyses

Data were imported and analysed using SPSS Version 25 (IBM Corporation, Armonk, NY, USA). Demographic data for the group as a whole, and for podiatrists and physical therapists separately, were described using mean (standard deviations; SD), median (inter-quartile range; IQR) or number (percentage), as appropriate. Chi-square tests were used to investigate differences between podiatrists and physical therapists. Where associations were found, standardised residuals were examined to identify sources of significant differences.

## Results

### Sample characteristics

Two hundred respondents (*n* = 113 (57%) podiatrists and *n* = 140 (70%) from Australia) completed the survey. The clinical and demographic characteristics of the sample are displayed in Table 2. There were slightly more females and podiatrists in the sample, and the group had an average of nearly 15 years of clinical experience. Characteristics of the podiatrist and physical therapist clinician subgroups were generally similar, although there were more physical therapist respondents than podiatrists from the UK, and there were more podiatrists than physical therapists in the 40–49 years age range. More podiatrists than physical therapists reported treating patients with first MTP joint OA frequently (28% vs 16%, *P* < 0.001) and very frequently (51% vs 1%, *P* < 0.001) and fewer reported treating these patients infrequently (2% vs 26%, *P* < 0.001) and somewhat frequently (19% vs 56%, *P* < 0.001). More physical therapists had undertaken postgraduate training in exercise therapy (51% vs 23%, *P* = 0.000) and manual therapy (64% vs 37%, *P* = 0.001), whilst more podiatrists had undertaken postgraduate training in pharmacology (21% vs 7%, *P* = 0.02) and orthotic/footwear prescription (55% vs 23%, *P* < 0.001).

**Table 2:** Clinical and demographic characteristics of respondents. Values are expressed as Number (%) unless reported otherwise (*n* = 200)

	Total Sample (*N* = 200)	Podiatrists (*N* = 113)	Physical therapists (*N* = 87)	*p*-value
Country				< 0.01
Australia	140 (70)	92 (81)	48 (55)	
United Kingdom	60 (30)	21 (19)	39 (45)	
Sex, female	117 (59)	61 (54)	56 (64)	0.14
Profession				NA
Podiatrist	113 (57)	113 (100)	0 (0)	
Physical therapist	87 (44)	0 (0)	87 (100)	
Age range				< 0.01
29 years or younger	74 (37)	33 (29)	41 (47)	
30–39 years	54 (27)	25 (22)	29 (33)	
40–49 years	41 (21)	31 (27)	10 (12)	
50–59 years	28 (14)	21 (19)	7 (8)	
60–69 years	3 (2)	3 (3)	0 (0)	
Years since graduating, mean (SD)	14.2 (10)	16.3 (10.7)	11.5 (8.2)	< 0.01
Work setting				0.24
Exclusively private	121 (61)	73 (65)	48 (55)	
Exclusively public	36 (18)	16 (14)	20 (23)	
Combination	43 (22)	24 (21)	19 (22)	
Frequency treating patients with first MTP joint OA				< 0.01
Infrequently (≤1 in the last 6-months)	25 (13)	2 (2)	23 (26)	
Somewhat frequently (between 2 and 5 in the last 6-months)	70 (35)	21 (19)	49 (56)	
Frequently (at least 1 per month)	46 (12)	32 (28)	14 (16)	
Very frequently (at least 1 per week)	59 (30)	58 (51)	1 (1)	
Postgraduate training for osteoarthritis	60 (30)	32 (28)	28 (32)	0.55
Postgraduate training for foot pain	91 (46)	55 (49)	36 (41)	0.30
Postgraduate training in exercise therapy	70 (35)	26 (23)	44 (51)	< 0.01
Postgraduate training in manual therapy	98 (49)	42 (37)	56 (64)	< 0.01
Postgraduate training in pharmacology	27 (14)	21 (19)	6 (7)	0.02
Postgraduate training in orthotic/footwear prescription	82 (41)	62 (55)	20 (23)	< 0.01

Please note, total percentage values (calculated by summing each sub-category for each column) may be greater than 100% due to rounding

*MTP* Metatarsophalangeal

*Indicates *P* < 0.001; ** indicates *P* < 0.01; *** *P* < 0.05

### Assessment tests

Assessment tests for the vignette patient with first MTP joint OA are listed in Table 3. In the cohort as a whole, most (*n* = 164, 82%) indicated that they would order some kind of investigation, predominantly an x-ray (*n* = 151, 92%), with few other investigations typically performed. Most (*n* = 141, 71%) said they would use a standardised performance or impairment measure, with the most common being first MTP joint range of motion, visual gait assessment, and a foot posture assessment. Half of the sample indicated that they would use a patient-reported questionnaire as part of the assessment, most frequently a visual analogue or numerical rating scale of pain.

**Table 3:** Approaches used to assess a typical patient with first MTP joint OA. Values are expressed as Number (%), where (%) indicates the proportion of respondents indicating they would use an approach from the given category

Approach category	Specific approach	Total Sample	Podiatrists	Physical therapists	*p*-value
Investigations		164 (82)	97 (86)	67 (77)	0.11
	Ultrasound	16 (10)	12 (12)	4 (6)	0.12
	X-ray	151 (92)	91 (94)	58 (87)	0.11
	Special imaging (e.g. MRI, CT or bone scan)	20 (12)	9 (9)	11 (16)	0.27
	Lab tests (e.g. inflammatory markers, rheumatoid factors)	54 (33)	24 (25)	30 (45)*	0.04
	Other	6 (4)	3 (3)	3 (5)	0.64
Standardised performance measures		141 (71)	75 (66)	66 (76)	0.15
	Heel raise	84 (60)	37 (49)	47 (71)*	0.01
	Jack’s test/Hubscher’s manoeuvre	66 (47)	55 (73)	11 (17)*	0.000
	Foot posture assessment	107 (76)	53 (71)	54 (82)	0.12
	Resting/neutral calcaneal standing position	87 (62)	51 (68)	36 (55)	0.10
	Lunge test	72 (51)	38 (51)	34 (52)	0.92
	First MTP joint range of motion	127 (90)	70 (93)	57 (86)	0.17
	Hallux strength	55 (39)	21 (28)	34 (52)*	0.004
	Visual gait assessment	115 (82)	63 (84)	52 (79)	0.43
	Other	11 (8)	4 (5)	7 (11)	0.38
Questionnaires		99 (50)	45 (40)	54 (62)*	< 0.00
	Foot Health Status Questionnaire	12 (12)	8 (18)	4 (7)	0.12
	Short Form 12 or 36	11 (11)	0 (0)	11 (20)*	0.001
	Foot Function Index	21 (21)	15 (33)	6 (11)*	0.007
	American Orthopedic Foot and Ankle Score Hallux, Metatarsophalangeal-Interphalangeal Scale	5 (5)	0 (0)	5 (9)	0.05
	Baltimore Painful Foot Score	1 (1)	0 (0)	1 (2)	0.36
	Visual analogue scale/numeric rating scale of pain	78 (79)	34 (76)	44 (82)	0.47
	Manchester Foot Pain and Disability Index	7 (7)	5 (11)	2 (4)	0.15
	Other	8 (8)	1 (2)	7 (13)	0.05
Weight assessment		149 (75)	86 (76)	63 (72)	0.55

Please note, percentage values for each sub-category is the percentage of respondents of the total responding for that category

*P < 0.01

Responses from podiatrists and physical therapists were similar, except more podiatrists indicated that they would perform Jack’s test/Hubscher’s manoeuvre (*P* < 0.001) and use the Foot Function Index (*P* < 0.007). More physical therapists indicated they would want a laboratory test performed on the patient (*P* = 0.007), and that they would use heel raise (*P* = 0.008) and hallux strength (*P* = 0.004) tests, and use the Short Form 12 or 36 questionnaire (*P* = 0.001).

### Treatment strategies

A range of treatment strategies were employed by the respondents to treat the vignette patient with first MTP joint OA (Table 4). In the entire cohort, the most common strategy was taping or padding, followed by exercise therapy, and hallux mobilisation/manipulation. Nearly all respondents indicated that they would offer advice, and that they would address analgesic use and the patient’s weight as part of the treatment.

**Table 4:** Therapist-administered, advice, analgesic and weight management approaches used to manage a typical patient with first MTP joint OA. Values are expressed as Number (%), where (%) indicates the proportion of respondents indicating they would use an approach from the given category

Approach category	Specific approach	Total Sample	Podiatrists	Physical therapists	*p*-value
Therapist-administered approaches	Any	200 (100)	113 (100)	87 (100)	NA
	Electrotherapy	13 (7)	2 (2)	11 (13)**	< 0.01
	Exercise therapy	117 (59)	38 (34)	79 (91)*	0.01
	Taping or padding	136 (68)	76 (67)	60 (69)	0.80
	Hallux manipulation/mobilization	99 (50)	41 (36)	58 (67)*	< 0.01
	Other joint manipulation/mobilization	45 (23)	14 (12)	31 (36)*	< 0.01
	Massage	45 (23)	11 (10)	34 (39)*	< 0.01
	Injection	23 (12)	15 (13)	8 (9)	0.37
	Trigger point techniques	33 (17)	11 (10)	22 (25)**	< 0.01
	Gait re-training	60 (30)	9 (8)	51 (59)*	< 0.01
	Stiffening shoe inserts	25 (13)	25 (22)	0 (0)*	< 0.01
	Footwear modifications	89 (45)	55 (49)	34 (39)	0.18
	Acupuncture	23 (12)	10 (9)	13 (15)	0.18
	Other	26 (13)	19 (17)	7 (8)	0.07
Advice approaches	Any	199 (99)	112 (99)	87 (100)	0.38
	Reducing activity levels	26 (13)	15 (13)	11 (13)	0.90
	Pacing of activities	126 (63)	54 (48)	72 (83)*	< 0.01
	Rest	44 (22)	24 (21)	20 (23)	0.77
	Nutrition	56 (29)	20 (18)	36 (41)*	< 0.01
	Use of walking aids	23 (12)	7 (6)	16 (18)**	0.01
	Use of heat/ice	143 (72)	74 (66)	69 (79)***	0.03
	Avoidance of painful movement/activities	66 (33)	44 (39)	22 (25)***	0.04
	Footwear advice	172 (86)	104 (92)	68 (78)**	0.01
	Other	38 (19)	23 (20)	15 (17)	0.58
Analgesia approaches	Any	174 (87)	98 (87)	76 (87)	0.90
	Advise to discuss with GP	135 (78)	69 (70)	66 (87)***	0.01
	Advise to discuss with pharmacist	49 (28)	22 (22)	27 (36)	0.06
	Discuss optimal usage of current medication	63 (36)	30 (31)	33 (43)	0.08
	Discuss/recommend over-the-counter medication	54 (31)	41 (42)	13 (17)*	< 0.01
	Discuss/recommend prescription medication	10 (6)	8 (8)	2 (3)	0.12
	Discuss use of alternative medicine (e.g. glucosamine, chondroitin sulphate)	54 (31)	38 (39)	16 (21)***	0.01
	Other	6 (3)	5 (5)	1 (1)	0.18
Weight management approaches	Any	160 (80)	85 (75)	75 (86)	0.05
	Advice about weight loss	98 (61)	42 (49)	56 (75)**	< 0.01
	Deliver a weight loss program	12 (8)	1 (1)	11 (15)**	< 0.01
	Refer to a dietician	68 (43)	34 (40)	34 (45)	0.50
	Refer to a weight loss program	32 (20)	14 (17)	18 (24)	0.24
	Other	35 (22)	26 (31)	9 (12)**	< 0.01

Please note, percentage values for each sub-category is the percentage of respondents of the total responding for that category

* *P* < 0.001; ** *P* < 0.01; *** *P* < 0.05

There were several differences in the treatments most commonly used by podiatrists and physical therapists to manage the vignette patient. For example, more podiatrists indicated that they would use shoe stiffening inserts (*p* < 0.001) and more would advise the patient on appropriate footwear (*p* = 0.005) and to avoid painful movements/activities (*p* = 0.04). In contrast, more physical therapists said they would use exercise therapy, hallux and other joint manipulation/mobilisation, massage, and gait retraining (all *p* < 0.001), and more would advise the patient on pacing of activities and nutrition (both *p* < 0.001) and on use of heat/ice (*p* = 0.032). More podiatrists indicated that they would discuss/recommend over-the-counter (*p* < 0.001) and alternative (*p* = 0.012) medications, while more physical therapists said they would advise the patient to discuss analgesia with their general practitioner (*p* = 0.010) and would provide advice about weight loss (*p* = 0.001).

Table 5 lists the most common exercise and orthotic treatments that podiatrists and physical therapists would use for the vignette patient. Flexibility/range of motion exercise was the most common response in the group as a whole, followed by intrinsic and extrinsic muscle strength exercises. More than three quarters of the sample/indicated that they would use a foot orthosis, with most using a custom-made device followed by a prefabricated device.

**Table 5:** Exercise and orthoses approaches used to manage a typical patient with first MTP joint OA. Values are expressed as Number (%), where (%) indicates the proportion of respondents indicating they would use an approach from the given category

Approach category	Specific approach	Total Sample	Podiatrists	Physical therapists	*p*-value
Exercise approaches	Any	170 (85)	86 (76)	84 (97)	< 0.01
	Local/intrinsic foot strengthening	112 (66)	43 (50)	69 (82)	< 0.01
	Lower limb/extrinsic foot strengthening	103 (61)	37 (43)	66 (79)	< 0.01
	Aerobic training	33 (19)	5 (6)	28 (33)	< 0.01
	Proprioception/balance	99 (58)	28 (33)	71 (85)	< 0.01
	Flexibility/range of movement	123 (72)	57 (66)	66 (79)	0.07
	Hydrotherapy	64 (38)	24 (28)	40 (48)	< 0.01
	Functional task training	33 (19)	5 (6)	28 (33)	< 0.01
	Pilates	27 (16)	10 (12)	17 (20)	0.13
	Increasing general activity	101 (59)	42 (49)	59 (70)	< 0.01
	Other	10 (6)	7 (8)	3 (4)	0.21
Orthotic devices	Any	166 (83)	109 (97)	57 (66)	< 0.01
	Over-the-counter	80 (48)	44 (40)	36 (63)	< 0.01
	Prefabricated/semi-custom	83 (50)	57 (52)	26 (46)	0.41
	Custom	109 (66)	85 (78)	24 (42)	< 0.01

Please note, percentage values for each sub-category is the percentage of respondents of the total responding for that category

*P < 0.001; ** *P* < 0.01; *** *P* < 0.05

There were a number of differences between the most common exercise and orthotic approaches used by podiatrists and physical therapists. For instance, more podiatrists indicated that they would prescribe an orthotic device (*p* < 0.001), and of those, most would prescribe custom orthoses (*p* < 0.001). In contrast, more physical therapists than podiatrists indicated that they would use exercise approaches (*p* < 0.001). In particular, more physical therapists responded that they would use intrinsic and extrinsic foot strengthening, aerobic training, and proprioception/balance exercise (all *p* < 0.001), as well as hydrotherapy (*p* = 0.008) and increasing general exercise (*p* = 0.004). Although fewer physical therapists than podiatrists indicated that they would prescribe orthoses, those that did mostly responded that they would prescribe an over-the-counter device (*p* = 0.005).

## Discussion

This is the first study to investigate how podiatrists and physical therapists in Australia and the UK assess and treat people with first MTP joint OA. Our findings show that an array of strategies are used by the respondents, with many differences apparent between the two professions. Specifically, podiatrists indicated that they used more footwear, orthotic and medication treatment strategies, while the physical therapists used more exercise- and manual therapy-based approaches.

The high use of imaging reported by respondents in our survey (94% of podiatrists and 87% of physical therapists) is a concern given that routine use of x-rays is not advocated in international OA guidelines [13], and contradicts recent recommendations which state that imaging is not required for a diagnosis in patients with a typical presentation of peripheral joint OA [14]. These findings are consistent with clinical practice patterns of Australian general practitioners, who also show a high use of diagnostic radiology in patients with foot [10], knee and hip OA [15]. The high rate of imaging identified in this study may be partly related to an absence of clinical diagnostic criteria for diagnosing OA at any foot joint [16], albeit radiographic OA of the first MTP joint can be accurately identified using simple clinical test [17]. In fact, the EULAR recommendations for the use of imaging in the clinical management of peripheral joint OA specifically noted that no studies have evaluated the impact of imaging for the management of foot OA [14]. Further work is needed to determine the diagnostic and clinical utility of imaging in first MTP joint OA. Likewise, there is also limited evidence supporting other assessment approaches used by respondents, such as Jack’s test [18] and the heel raise test [19]. Additional research is needed to determine whether these approaches aid clinicians in making an accurate diagnosis or for monitoring treatment effectiveness.

The difference in treatments used by podiatrists and physical therapists to manage people with first MTP joint OA is likely due to both the training and scope of practice of these two professions. For instance, podiatrists receive extensive undergraduate training in orthotic prescription and pharmacology, in addition to other treatment approaches, whereas physical therapists receive comparatively little training in these treatments, and instead receive more training in manual therapy and exercise techniques. Support for this is also found in the number of respondents from each profession that had undergone additional postgraduate education in these treatment strategies. Results of our survey show that more podiatrists reported postgraduate training in pharmacology and orthotic/footwear prescription, while more physical therapists reported postgraduate training in exercise and manual therapy.

There are few RCTs on management strategies for people with first MTP joint OA, making it difficult to comment on the evidence for, or against, these commonly used treatments. However, there is some evidence to support some of the approaches used by the two professions from our survey. For instance, in a parallel-group randomised comparative effectiveness trial, Menz and colleagues found clinically-meaningful improvements with both prefabricated foot orthoses and rocker-sole shoes in 102 people with first MTP joint OA [5]. Although improvements were of similar magnitude with both interventions, a greater proportion of participants in the foot orthoses group reported being at least moderately improved (a score of ≥ 4 on a 15-point Likert scale, where − 7 represents a very great deal worse and + 7 represents a very great deal better), and had greater adherence, and fewer adverse events. Other research has found the addition of sesamoid mobilisation, foot strengthening and gait training to other physical therapies, including whirlpool, ultrasound, stretching and cold packs, resulted in significantly greater improvements in pain, range of motion and strength. However, the small sample (*n* = 20) and lack of an adequate control in this study limits the generalisability of these findings.

Indirect support for exercise as a management approach for first MTP joint OA may be found in clinical OA guideline recommendations that are largely based on evidence from knee and hip OA. Exercise, in addition to self-management strategies such as advice regarding physical activity, weight loss and analgesia, is recommended as a core treatment for knee and hip OA in most national (e.g. [20, 13]) and international (e.g. [21, 22]) clinical OA guidelines. Furthermore, greater physical activity such as walking, may not necessarily exacerbate pain given recent research showed cumulative plantar stress, calculated as the product of plantar pressures and mean steps per day, was not associated with first MTP joint pain in a large community based cohort sample [23]. However, given the unique structure and biomechanical function of the first MTP joint, we cannot necessarily extrapolate from the studies that informed these guidelines that exercise is similarly effective for first MTP joint OA. Future research is needed to investigate whether exercise strategies reported by podiatrists and physical therapists in this study, such as general physical and targeted strength and range of motion exercises, are effective for treating symptoms associated with first MTP joint OA.

There are some limitations to our study that should be considered when interpreting our results. Firstly, we do not know how many eligible podiatrists and physical therapists in Australia and the UK our survey reached therefore we were unable to calculate the response rate. Furthermore, the proportion of UK podiatrists who competed our survey was also relatively low and it is possible that this may have influenced our findings. Unlike podiatrists in Australia, podiatrists in the UK also have prescription-only medication training at the undergraduate level. This difference in training between countries may have affected responses to the question on postgraduate training in pharmacology. Finally, we only surveyed podiatrists and physical therapists from these two countries, thus our findings may not be generalisable to other health professions in these two countries, or to these two professions in other countries.

## Conclusion

In conclusion, Australian and UK podiatrists and physical therapists utilise an array of assessment and treatment strategies to manage people with first MTP joint OA, although the clinical utility of some of these approaches is uncertain. Footwear, foot orthoses and medication were used more often by podiatrists, whereas exercise-based approaches were used more often by physical therapists. Although many of the approaches used by both professions are recommended in OA clinical practice guidelines, these recommendations are usually based on evidence from people with OA at other sites. Further research is needed to determine whether these commonly-used approaches improve symptoms associated with OA of the first MTP joint.

## Data Availability

The datasets used and/or analysed during the current study are available from the corresponding author on reasonable request.

## Abbreviations

IQR: Interquartile range
MTP: Metatarsophalangeal
N: Number
OA: Osteoarthritis
RCT: Randomised controlled trial
SD: Standard deviation
UK: United Kingdom

## References

[1] Pereira D, Peleteiro B, Araújo J, Branco J, Santos RA, Ramos E (2011). The effect of osteoarthritis definition on prevalence and incidence estimates: a systematic review. Osteoarthr Cartil.

[2] Roddy E, Thomas MJ, Marshall M, Rathod T, Myers H, Menz HB (2015). The population prevalence of symptomatic radiographic foot osteoarthritis in community-dwelling older adults: cross-sectional findings from the clinical assessment study of the foot. Ann Rheum Dis.

[3] Bergin SM, Munteanu SE, Zammit GV, Nikolopoulos N, Menz HB (2012). Impact of first metatarsophalangeal joint osteoarthritis on health-related quality of life. Arthritis Care Res.

[4] Munteanu SE, Zammit GV, Menz HB, Landorf KB, Handley CJ, Elzarka A (2011). Effectiveness of intra-articular hyaluronan (Synvisc, hylan G-F 20) for the treatment of first metatarsophalangeal joint osteoarthritis: a randomised placebo-controlled trial. Ann Rheum Dis.

[5] Menz HB, Auhl M, Tan JM, Levinger P, Roddy E, Munteanu SE (2016). Effectiveness of foot orthoses versus rocker-sole footwear for first metatarsophalangeal joint osteoarthritis: randomized trial. Arthritis Care Res.

[6] Shamus J, Shamus E, Gugel RN, Brucker BS, Skaruppa C (2004). The effect of sesamoid mobilization, flexor hallucis strengthening, and gait training on reducing pain and restoring function in individuals with hallux limitus: a clinical trial. J Orthop Sports Phys Ther.

[7] Baumhauer JF, Singh D, Glazebrook M, Blundell C, De Vries G, Le IL (2016). Prospective, randomized, multi-centered clinical trial assessing safety and efficacy of a synthetic cartilage implant versus first metatarsophalangeal arthrodesis in advanced hallux rigidus. Foot Ankle Int.

[8] American Academy of Orthopaedic Surgeons (2017). Management of osteoarthritis of the hip: Evidence-based clinical practice guideline.

[9] Thomas MJ, Peat G, Rathod T, Marshall M, Moore A, Menz HB (2015). The epidemiology of symptomatic midfoot osteoarthritis in community-dwelling older adults: cross-sectional findings from the clinical assessment study of the foot. Arthritis Res Ther.

[10] Paterson KL, Harrison C, Britt H, Hinman RS, Bennell KL (2018). Management of foot/ankle osteoarthritis by Australian general practitioners: an analysis of national patient-encounter records. Osteoarthr Cartil.

[11] Hughes R, Huby M (2002). The application of vignettes in social and nursing research. J Adv Nurs.

[12] Rutten GM, Harting J, Rutten ST, Bekkering GE, Kremers SP (2006). Measuring physiotherapists’ guideline adherence by means of clinical vignettes: a validation study. J Eval Clin Pract.

[13] National Clinical Guideline Centre (2014). Osteoarthritis: Care and management in adults. Clinical guideline CG177. Methods, evidence and recommendations.

[14] Sakellariou G, Conaghan PG, Zhang W, Bijlsma JWJ, Boyesen P, D'Agostino MA (2017). EULAR recommendations for the use of imaging in the clinical management of peripheral joint osteoarthritis. Ann Rheum Dis.

[15] Brand CA, Harrison C, Tropea J, Hinman RS, Britt H, Bennell K (2014). Management of osteoarthritis in general practice in Australia. Arthritis Care Res.

[16] Paterson KL, Gates L (2019). Clinical assessment and Management of Foot and Ankle Osteoarthritis: a review of current evidence and focus on pharmacological treatment. Drugs Aging.

[17] Zammit GV, Munteanu SE, Menz HB (2011). Development of a diagnostic rule for identifying radiographic osteoarthritis in people with first metatarsophalangeal joint pain. Osteoarthr Cartil.

[18] Halstead J, Redmond AC (2006). Weight-bearing passive dorsiflexion of the hallux in standing is not related to hallux dorsiflexion during walking. J Orthop Sports Phys Ther.

[19] Nawoczenski DA, Baumhauer JF, BRJJ U (1999). Relationship between clinical measurements and motion of the first metatarsophalangeal joint during gait. JBJS.

[20] Royal Australia College of General Practitioners (2017). Guideline for the management of knee and hip osteoarthritis. Consultation draft.

[21] Fernandes L, Hagen KB, Bijlsma JWJ, Andreassen O, Christensen P, Conaghan PG (2013). EULAR recommendations for the non-pharmacological core management of hip and knee osteoarthritis. Ann Rheum Dis.

[22] McAlindon TE, Bannuru RR, Sullivan MC, Arden NK, Berenbaum F, Bierma-Zeinstra SM (2014). OARSI guidelines for the non-surgical management of knee osteoarthritis. Osteoarthr Cartil.

[23] Rao S, Douglas Gross K, Niu J, Nevitt MC, Lewis CE, Torner JC (2016). Are pressure time integral and cumulative plantar stress related to first metatarsophalangeal joint pain? Results from a community-based study. Arthritis Care Res.

